# Temperature effects on ballistic prey capture by a dragonfly larva

**DOI:** 10.1002/ece3.3975

**Published:** 2018-04-02

**Authors:** Estefania Quenta Herrera, Jérôme Casas, Olivier Dangles, Sylvain Pincebourde

**Affiliations:** ^1^ Institut de Recherche sur la Biologie de l'Insecte UMR 7261, CNRS Université de Tours, Tours France; ^2^ Institut de Recherche pour le Développement (IRD) UMR EGCE‐Université Paris Sud‐CNRS‐IRD‐Paris Saclay Gif‐sur‐Yvette France

**Keywords:** attack velocity, capture success, climate change, escape velocity, predator–prey interaction, speed–accuracy trade‐off, temperature

## Abstract

Understanding the effects of temperature on prey–predator interactions is a key issue to predict the response of natural communities to climate change. Higher temperatures are expected to induce an increase in predation rates. However, little is known on how temperature influences close‐range encounter of prey–predator interactions, such as predator's attack velocities. Based on the speed–accuracy trade‐off concept, we hypothesized that the increase in predator attack velocity by increasing temperature reduces the accuracy of the attack, leading to a lower probability of capture. We tested this hypothesis on the dragonfly larvae *Anax imperator* and the zooplankton prey *Daphnia magna*. The prey–predator encounters were video‐recorded at high speed, and at three different temperatures. Overall, we found that (1) temperature had a strong effect on predator's attack velocities, (2) prey did not have the opportunity to move and/or escape due to the high velocity of the predator during the attack, and (3) neither velocity nor temperature had significant effects on the capture success. By contrast, the capture success mainly depended on the accuracy of the predator in capturing the prey. We found that (4) some 40% of mistakes were undershooting and some 60% aimed below or above the target. No lateral mistake was observed. These results did not support the speed–accuracy trade‐off hypothesis. Further studies on dragonfly larvae with different morphological labial masks and speeds of attacks, as well as on prey with different escape strategies, would provide new insights into the response to environmental changes in prey–predator interactions.

## 1 INTRODUCTION

The interaction between prey and predators is a key component in determining the flux of nutrients from individuals to ecosystems (Thompson et al., 2012). Understanding how temperature influences the different processes of prey–predator interactions is an important step toward anticipating potential community‐ and ecosystem‐level impacts of warming. Warming has profound but complex effects on interaction strength between ectothermic prey and predators (Pincebourde, Sanford, Casas, & Helmuth, 2012; Rall, Vucic‐Pestic, Ehnes, Emmerson, & Brose, 2010). Nevertheless, increasing temperature within a tolerable range is expected to increase predation rates because it enhances the metabolism of ectothermic species (Brown, Gillooly, Allen, Savage, & West, 2004). This expectation was verified by numerous empirical studies, all illustrating the diversity of mechanisms at play (e.g., Jeanne, 1979; Sanford, 1999; Vucic‐Pestic, Ehnes, Rall, & Brose, 2011). For example, temperature influences the movement and behavior of predators (Dell, Pawar, & Savage, 2014; Öhlund, Hedström, Norman, Hein, & Englund, 2015). Predators move faster at higher temperatures while they are searching for prey, thereby increasing the contact with their resource and leading to higher capture rates (Dell et al., 2014; Sentis, Hemptinne, & Brodeur, 2013). Additionally, temperature increase makes predators more efficient at handling prey, resulting in shorter times for attacking, killing, and digesting prey (Dreisig, 1981; Sentis, Morisson, & Boukal, 2015; Sentis et al., 2013). Elevated temperature can also modify the emission of chemical signals by the predator, inducing thereby a change in the prey' escape responses when the predator is present (Mondor, Tremblay, Awmack, & Lindroth, 2004; Sentis, Ramon‐Portugal, Brodeur, & Hemptinne, 2015).

Predation involves a series of steps: prey encounter, prey detection, attack and consumption, and different strategies arise during those steps (Brechbühl, Casas, & Bacher, 2011; Endler, 1991). Some predators move fast to capture their prey, thereby overcoming the warning and escape system of the prey, even if predators are highly conspicuous (e.g., Casas & Steinmann, 2014). The accuracy of predators during fast attacks and the prey escape strategy might be major determinants for the capture success and/or failure of the predator (Brechbühl et al., 2011; Soto, Stewart, & McHenry, 2015). Based on this general background, temperature can be expected to modulate the relative velocities of attack and escape of ectothermic predators and prey, thereby altering the strategies of the individuals during the prey–predator interaction. For instance, the increase in predator attack velocity at higher temperature might reduce its accuracy, leading to more opportunities for the prey to escape.

This effect has been called the speed–accuracy trade‐off which states that faster choices reduce precision, whereas slower choices are highly accurate (Chittka, Dyer, Bock, & Dornhaus, 2003). This hypothesis was verified with animals in various contexts, including decision‐making ants under windy (harsh) conditions (Franks, Dornhaus, Fitzsimmons, & Stevens, 2003), foraging bees (Ings & Chittka, 2008), decision making in zebrafish according to their “personality” (Wang, Brennan, Lachlan, & Chittka, 2015) or foraging decision in an unicellular slime (Latty & Beekman, 2011). To our knowledge, however, this theoretical background was widely applied to predator–prey interaction only in one system: Foraging bees slow their inspection flights over a flower after learning from pheromones emitted by conspecifics that there is a risk from cryptic spiders (Ings & Chittka, 2008). A longer inspection effort results in accurate predator detection, thereby decreasing predation risk at the expense of foraging time. In this example, the prey is mobile while the predator (crab spider) has a sit‐and‐wait strategy. In this case, the speed–accuracy trade‐off was applied therefore only to the prey. However, the speed–accuracy trade‐off was poorly investigated in the inverse situation, that is, when the predator moves fast and/or make fast choices to grab the prey (e.g., Chang, Ng, & Li, 2017). Here, we investigated the speed–accuracy trade‐off in a predator with a fast attack strategy when capturing a prey with a slow or immobile reaction during the predator's attack. Specifically, we tested the hypothesis that increasing temperature speeds up the attack of the predator but at the expense of its accuracy in grabbing the prey.

We used the dragonfly larvae *Anax imperator* (Odonata: Aeshnidae) and the zooplankton *Daphnia magna* (Diplostraca: Daphniidae) as a predator–prey system to understand the potential effects of temperature on the predator strategy during their interaction. *Daphnia* is an important part of the diet of Aeshnidae larvae (e.g., Blois, 1985). Dragonfly larvae capture their prey by extending at a high velocity their labial mask which has movable hooks (Pritchard, 1965). This type of attack is called ballistic and is similar to tongue projection in chameleons and frogs. Two mechanisms have been suggested to explain the functioning of the labial mask (Olesen, 1972; Parry, 1983; Pritchard, 1986; Tanaka & Hisada, 1980). First, the abdominal contraction generates hemolymph pressure up to the labial mask, causing its extension. Second, the primary flexor muscle in the labial mask allows the extension of the mask, and also potentially prevents the labial extension during the jet propulsion. For the prey, two strategies were described for *Daphnia,* a “hop‐and‐sink” and a “zooming” behavior. Both types of locomotion create rapid vertical accelerations but differ in that the “zooming” behavior does not have a passive sinking phase (CO'Keefe, Brewer, & Dodson, 1998). Furthermore, *Daphnia* swims up more actively when temperature rises, and it tends to sink when temperature drops (Gerritsen, 1982). However, given the time needed for *Anax* larvae to fully extend their labial mask (range 25–40 ms; Tanaka & Hisada, 1980) compared to the average swimming velocities of *Daphnia* (ranging from 0.0035 to 0.015 m/s, Baillieul, De Wachter, & Blust, 1998; Porter, Gerritsen, & Orcutt, 1982; Weber & Van Noordwijk, 2002), we determined that the *Daphnia* can move over a distance of <1 mm at best during the predator attack, which is much less than the distance between the two hooks of the labial mask (about 5 mm; Parry, 1983). Therefore, the prey remains nearly immobile during the predator attack. We quantified the velocities and the predator capture success under different temperatures by analyzing sequences of images obtained with a high‐speed video camera. Although we focused on the predator kinematics, we also verified that the prey was almost immobile during the interaction to ensure that the level of inaccuracy of the predator was due to the speed–accuracy trade‐off and not to the movement of the prey.

## 2 MATERIALS AND METHODS

### 2.1 Experimental design

Larvae of the predator *A. imperator* (Aeshnidae) were captured in the Rillé lake in France (47°28′12.00″N, 0°12′21.60″E). They were placed in separated boxes of 10 × 13 × 4.5 cm with 250 ml of dechlorinated water and kept in a room at 20°C and photoperiod 14:10 light–dark. They were fed with zooplankton, worms, and mosquito larvae twice a week until the experiments began. The prey *D. magna* (Daphnidae) was obtained from an aquaculture company (Aqualiment, Niederbronn Les Bains, France). It was reared in the laboratory at 20°C with a photoperiod of 14:10 light–dark, and it was fed with *Chlorella* sp. The body size of the predators varied among them (mean = 3.6 cm, min = 2.31 cm, max = 4.30 cm). The body size of the prey was also slightly variable (mean length = 2.25 mm, min = 1.55 mm, max = 2.8 mm). The body size of organisms was measured with an image analyzer program (ImageJ software, v1.5p, National Institutes of Health, USA) from the videos.

The prey–predator close encounters were video‐recorded by putting boxes each containing one dragonfly larva and one *Daphnia* inside a water bath (heating and cooling) to manipulate the temperature. During the experiments, sand and aquatic plants were added to the boxes to reduce the stress of the individuals. The parameters of the prey–predator interaction were determined at three temperature treatments (15, 20, and 30°C). For each treatment, four replications (i.e., four different individuals) were carried out (total *N* = 12 predator individuals). One individual larva died during the experiment. Dissolved oxygen (DO_2_) was unrelated to the temperature because the boxes were relatively small (mean DO_2_ was 7.6 and 7.4 mg/L at 20 and 28°C, measured using a probe U26‐001, Hobo, Prosensor, France). Before the experiment, individuals were acclimated at the corresponding temperature for 1 day in a climatic chamber. This acclimation period reflects roughly natural conditions: Field recordings showed that water temperature cycles over a period of 24 hr (unpublished data). Movements of the predator and the prey during the interaction were recorded by analyzing images obtained from a high‐speed video camera (Vision Research Phantom V9.0). The camera was positioned perpendicular to the horizontal (above the water bath) to record the *x*,* y* coordinates of the movement. Two filming speeds were used, 2,000 and 100 images per second. Both were used because an increase in the number of images per second decreases the filming period. Thus, with the first speed, we observed precisely the movement of the labial mask of the predator and the type of predator failure to capture the prey. With the second speed, we obtained longer films to determine the number of attacks of the predator. A minimum of four films were recorded for each individual. We selected only those films with the best quality of recording, and with the labial mask of the predator positioned in a flat position. Thus, a total of 42 films were selected for the high‐velocity group of films (i.e., 13 films at 15°C, 17 at 20°C and 12 at 30°C; see Table S1). Similarly, 54 films were obtained with the low‐speed camera (i.e., 14 films at 15°C, 23 at 20°C and 17 at 30°C; see Table S2), of which, we selected 33 films. Therefore, a total of 75 films (i.e., 42 + 33 films) were used to analyze the predator fail types during a single attack, and 54 to measure the number of attacks over longer periods. The recording for the number of attacks (54 films) started at the initial moment of predator's first attack, and stopped when the predator gave up ostensibly (see details of the experimental design in Appendix S1: Tables S1 and S2).

### 2.2 Data analysis

**Figure 1:**
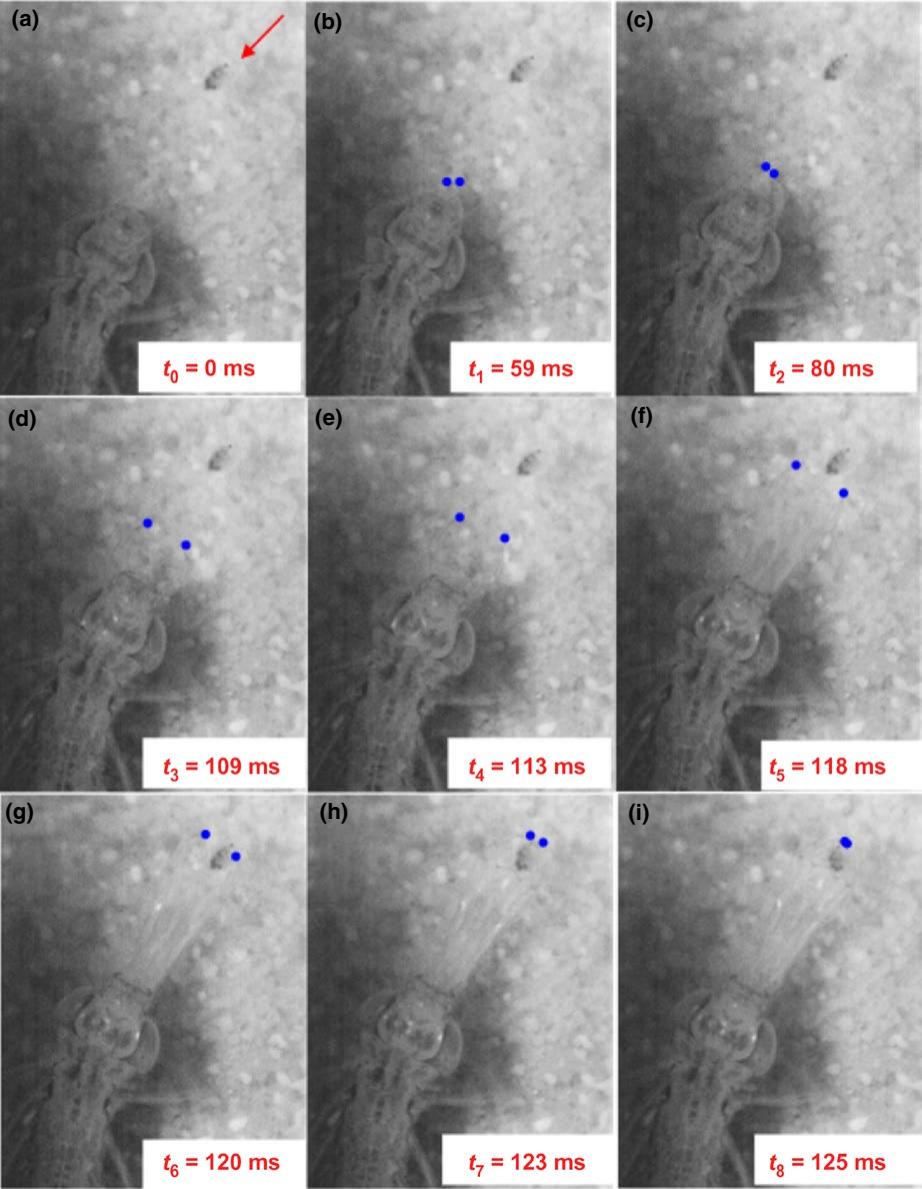
Dragonfly larva *Anax imperator* attacking its zooplankton prey *Daphnia magna*. Blue points represent the end of the two hooks of the labial mask of the predator at different times, from 0 to 125 ms. Images from (a) to (d) represent the initial movement of the labial mask. Images from (e) to (i) represent the terminal movement of the labial mask. In this example, the first phase is slow, taking 109 ms to reach position (d). The second phase is much faster (16 ms). The arrow in (a) indicates the position of the Daphnia. In this case, *Daphnia magna* did not move during the attack

To assess the impact of temperature on predator's movements, four metrics were calculated. First, two global velocities were determined as follows: the average velocity of the attack (when the labial mask extends forward), and the average velocity of the return (when the labial mask returns after the attack). Subsequently, the displacement of the attack was analyzed from the (*x*,* y*) coordinates of the labial mask position using the ImageJ software. A total of 42 cumulative curves of distance vs. time were obtained (Figure 1; Appendix S2). Two phases with different velocities were observed during the extension of the labial mask, called initial and final velocities, which represent the initial and terminal phases, respectively (Figure 1; Appendix S2). During the initial phase, the larva sets the labial mask by slowly adjusting its position. The mask is then extended forward at high velocity during the terminal phase. The initial and final velocities of these two phases of the predator's attack were estimated by fitting linear models on the cumulative relationship between distance and time, with the broken‐line method and using the *segmented* R package (Muggeo, 2008). Before the fitting, we confirmed visually that the movement of the individuals had the same pattern through the standardization of the curves, by dividing by their maximum value (Appendix S2). The effect of temperature on the four types of velocities was analyzed with mixed linear regression models (LMM), using individuals as a random factor to take into account the individual predator variability, with *lme4* R package (Bates, Mächler, Bolker, & Walker, 2015). Because predator size might be a major factor in determining velocity, we analyzed the effect of body, labial mask, and abdomen sizes, and their interactions with temperature. No significant effect of these morphological characteristics was observed (see Appendix S3). Furthermore, we analyzed the effect of temperature on the number of attacks using a mixed linear regression analysis with the same approach as above.

Prey did not show any movement during the attack of the predator, except in two cases only. Thus, the prey was considered immobile relative to the predator whatever the temperature. To analyze the effect of temperature on the capture success of the predator, we performed generalized mixed models with a binomial distribution (GLMM). Because the capture success of the predator can be influenced by factors such as the distance between the prey and the predator or the velocity of the predator, we tested nine models with three independent variables: temperature, distance, velocities, and their interactions. The parameters of the fits were assessed by the maximum likelihood using Laplace approximations, chi‐squared tests, and the Akaike information criterion (AIC), following the procedures of Bates et al. (2015) and Bolker et al. (2009), and using the *lme4* package in R statistical software vs 3.4.0 (Bates et al., 2015).

## 3 RESULTS

**Figure 2:**
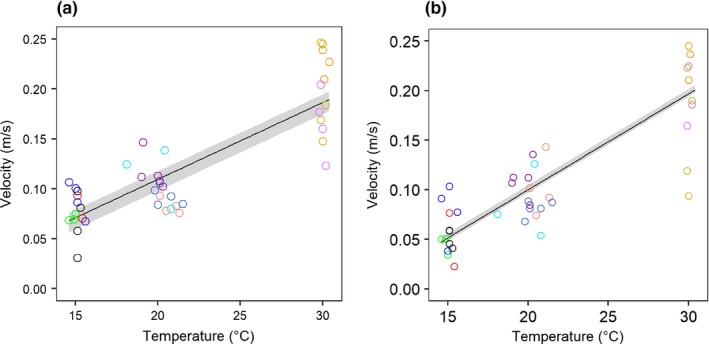
Temperature effects on average velocities of the predator's attack: (a) when the labial mask moved forward, (b) when the labial mask returned to its initial position. Different colors indicate the 11 different individuals. The shade of gray gives the 95% interval of the linear regression

**Figure 3:**
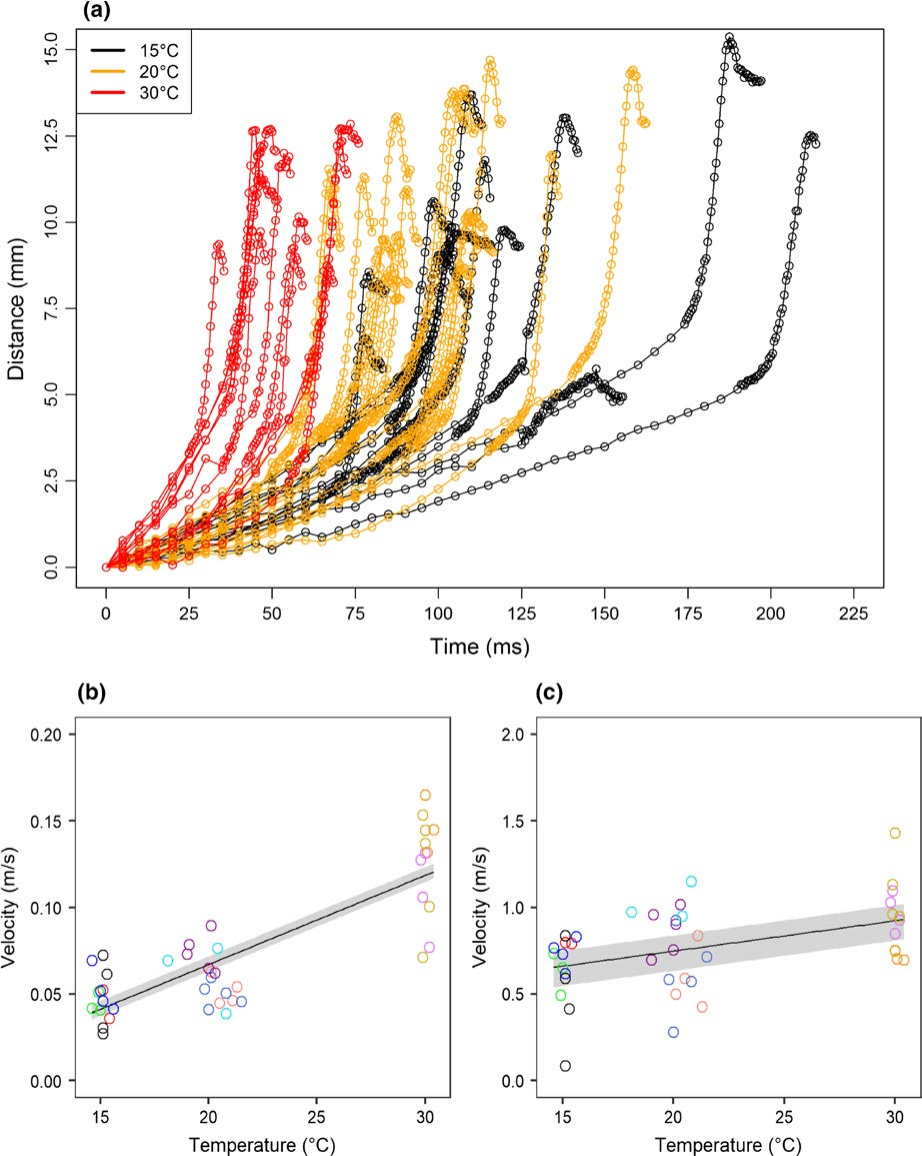
(a) Distance travelled by the labial mask of the predator over time during the attack as function of temperature. (b and c) The effect of temperature on the two velocities (b‐initial, and c‐final) of predator's attack. Different colors indicate the 11 different individuals. The gray shade gives the 95% interval of the linear regression. Note the 10‐fold scale difference between the two velocities

Temperature had a positive effect on the average velocities of the predator's attack, both when the labial mask moved forward and when it returned to its initial position (χ^2^ = 17.98, *p* < .001, χ^2^ = 26. 89, *p* < .001, Figure 2a,b; Appendix S4). A separate analysis further revealed that temperature influenced both the initial and the final velocities (χ^2^ = 18.78, *p* < .001, χ^2^ = 4.26, *p* < .05, Figure 3a–c; Appendix S4). The effect of temperature on the velocity of the initial (slow) phase was much higher than the effect on the velocity of the terminal (fast) phase. There was no significant effect of temperature on the number of attacks (χ^2^ = 1.10, *p* = .29, see Appendix S5).

The prey did not manifest any movement during the attack of the predator (except in two observations), and capture was successful in 61.3% of the 75 cases. However, in 38.6% of the observations, the predator failed to capture the prey. Four types of predator failure (to capture the prey) were categorized (Table 1). In only two cases (of the 75 observations), the prey slightly moved at the same time of the labial mask or when the predator closed its movable hooks in only two instances (escape#1 and escape#2). The prey was not captured due to the inaccuracy of the predator in the vertical axis in 55.1% of the total number of failures. In this case, the position of the prey was above or below the labial mask of the predator. We did not observe lateral mismatches. In 37.9% of the number of failures, the prey was not captured due to undershooting of the predator (i.e., the distance to the prey was larger than the maximal distance reached by the labial mask).

**Table 1:** Types of failures of the predator *Anax imperator* when attacking the prey *Daphnia magna* in the total of 75 observations

Type of failure	Number of cases	% (Total = 75)	% (Total = 29)	Details
Escape#1: prey movement	1 (at 15.1°C)	1.33	3.44	Prey moved at the same time as the labial mask of the predator
Escape#2: prey movement	1 (at 29.8°C)	1.33	3.44	Prey moved when the predator closed their movable hooks to capture it
Failure#1: predator inaccuracy	11 (3 at 15°C, 6 at 20°C, 2 at 30°C)	14.67	37.93	Prey escape after the attack of the predator, due to undershooting
Failure#2: predator inaccuracy	16 (6 at 15°C, 7 at 20°C, 3at 30°C)	21.33	55.17	Prey escape due to the inaccuracy of the predator in the vertical axis
Total	29	38.6	100	

Among the four types, we discriminated two types of escape due to prey movement and two types of failure due to the inaccuracy of the predator. The proportion relative to the number of escapes (*N* = 29) is also shown.

**Figure 4:**
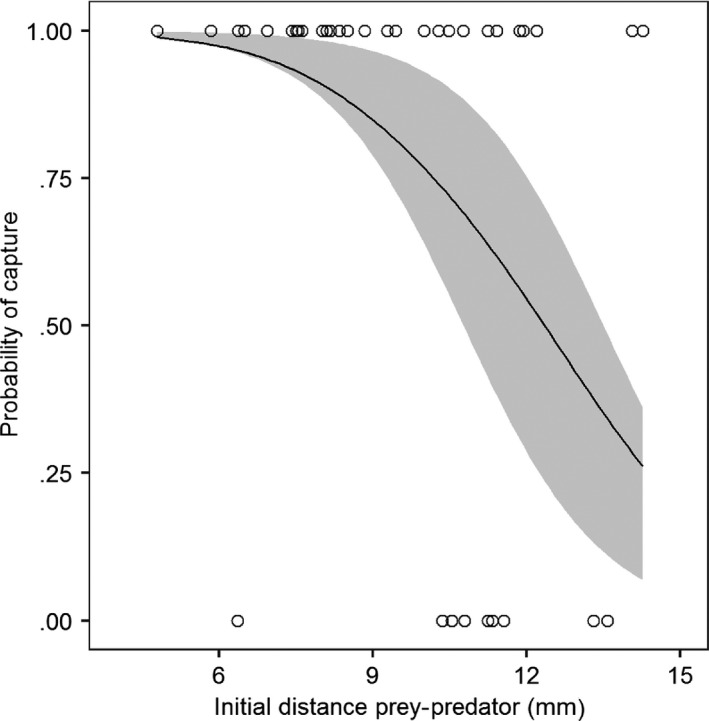
Relationship between the probability of capture and the initial distance between the prey and the predator. Circles are observations. The shade portion represents the 95% confidence interval of the generalized linear mixed model

Generalized mixed models did not identify any significant effects of temperature and velocities on the capture success of the predator (see Appendix S6). Only the initial distance between the predator and the prey was significant (β_0_ = 5.43, β_1_ = −0.74, χ^2^ = 5.31, *p* < .05, Figure 4; Appendix S6). There is more than 50% of capture probability when the initial distance between the predator and the prey was about 12 mm, and the capture success probability started to drop when the distance was about 8 mm (Figure 4).

## 4 DISCUSSION

The effect of temperature on predator–prey interactions generally varies in its magnitude because the level of influence of temperature depends on the strategies used by both partners as well as their physiology (Vucic‐Pestic et al., 2011). In our predator–prey system, temperature did not influence the probability of inaccuracy of the predator, most likely because the strategy of the predator is to launch an extremely fast attack. A comparison of velocity values of the predator in our study with the swimming velocity of *Daphnia* shows that *A. imperator* is more than 10 times faster than *Daphnia*, with striking velocities ranging from 0.03 to 0.25 m/s. By contrast, the average swimming velocities measured over a small straight movement of five individuals of *Daphnia*, as measured from our films outside an attack phase (using the same methodology than for the labial mask velocity of the predator), ranged from 0.006 to 0.05 m/s, which is consistent with the average swimming velocities of *Daphnia* reported in the literature (ranging from 0.0035 to 0.015 m/s, Baillieul et al., 1998; Porter et al., 1982; Weber & Van Noordwijk, 2002). While escape movements seem unlikely given the difference in velocity between the predator and the prey, an intrinsic morphological propriety—the thickness of the shell—is a good strategy against predators in crustaceans (Brönmark, Lakowitz, & Hollander, 2011). In only one case, the prey was captured and escaped as the predator started to consume it. In this case, it is possible that the carapace of the prey provided an effective protection against the movable hooks of the predator (Laforsch, Ngwa, Grill, & Tollrian, 2004; Rabus, Söllradl, Clausen‐Schaumann, & Laforsch, 2013). Indeed, several studies showed that the morphological defenses in *Daphnia* (e.g., cuticle highly laminated, head spines) are key mechanisms against the predator's mouthparts (Sandusky & Deban, 2012). However, it is not known whether water temperature can influence this defense strategy by causing changes in cuticle toughness for instance.

The predator's strike was successful in more than half of the observations. A non‐negligible number of failures (~40%) were, however, recorded, and the prey was not captured due to the inaccuracy of the predator (i.e., wrong assessment of distances and the prey position in the vertical axis). These results support the hypothesis that the precision of the attack might be a key factor for the capture success in predator–prey systems in which fast predators are involved (Chittka et al., 2003; Soto et al., 2015). However, we did not find a significant effect of mask velocity on the capture success of the predator. The failures of the predator might be explained by other mechanisms, such as a wrong assessment of the angle at the start of the fast movement, the small body size of the prey which might be difficult to capture, or the erratic movement of the prey before the attack. This latter mechanism could cause a confusion and distraction of the predator, leading to higher survival chances for the prey (Humphries & Driver, 1970; Milinski, 1977). The type of movement of *Daphnia* depends on temperature—it reduces velocity and avoids instantaneous changes in velocity and direction, supposedly as an antipredator strategy (so‐called “fluid‐mechanical camouflage”; Ziarek, Nihongi, Nagai, Uttieri, & Strickler, 2011). To our knowledge, it is not known whether temperature can also influence the neuronal ability of dragonfly larvae to perceive visually its prey and assess their position.

Furthermore, the degree of mistakes by the dragonflies (about 40%) is similar to the rate of mistakes observed elsewhere with *Aeshna* preying on *Daphnia* (Hirvonen & Ranta, 1996) or dummies (Pritchard, 1965). How these mistakes are partitioned is rather constant among studies. We found that some 40% of mistakes were undershooting and some 60% aimed below or above the target. No lateral mistake was observed. Baldus (1926) has no quantitative data but mentions that undershooting is very rare and that these are usually near‐misses. Pritchard (1965) mentions that among the clear missed attacks he observed, some 25% were undershooting, the rest being targeted below the dummy, so in the vertical plane, and without any observation of lateral mistakes.

The behavior and the neuroethology of dragonfly larvae can explain the different types of mistakes (Baldus, 1926). The exact geometry of the eyes has been studied and eyes experimentally covered by Baldus (1926) and later authors: Before launching an attack, the dragonfly larva positions itself so that the prey is in the median axis of the predator's body. The position of the prey on the points of intersection of the ommatidia axes of the dragonfly larva is what determines whether the prey is caught or not. This therefore explains the lack of lateral mistakes. Errors in the vertical plane might be explained by the fact that libelluloid larvae have large palps that allow for a certain degree of error in the judgment of the direction of the prey. We do not have good explanation for undershooting, even more so as Pritchard (1965) mentions that the labium is usually extended further than the distance of the prey, ensuring no failure due to undershooting. The size of the prey, which was small relative to the predator in our experiments, may require a high precision level with the labial mask finishing its course exactly at the position of *Daphnia*. Indeed, the viscous forces that apply to an aquatic organism are higher for small sized bodies relative to large bodies (Denny, 2015). Therefore, small prey may be displaced by the flow generated by the extending labial mask due to these viscous forces. Such process was shown to cause small juvenile fishes to be food deprived despite the presence of an abundant food in the environment: The prey escaped fishes due to the viscous forces (China & Holzman, 2014; China, Levy, Liberzon, Elmaliach, & Holzman, 2017). Viscosity depends on water temperature, and therefore, we expect this phenomenon to be modulated by the interaction between prey size and temperature.

We did not find significant effects of temperature and velocity on the capture success of the predator. The predator accuracy was therefore independent of temperature. However, we found strong evidence for the effect of temperature on the predator's attack kinematics. The average velocity of the labial mask increases positively with temperature by a rate of 0.0076 mm/ms per 1°C of warming (see Appendix S3 for the other types of velocity). Studies on organisms with ballistic attacks (e.g., tongue projection in chameleons and frogs) suggested that these organisms generally have an elastic energy storage and/or muscle energy storages allowing them a very quick movement, relatively insensitive to temperature (Anderson & Deban, 2010; Deban & Lappin, 2011; Tanaka & Hisada, 1980). By contrast, we found that increasing temperature had an important effect on the velocity of the predator, which can be attributed to the effect of temperature on both the abdominal muscles and the primary flexor muscles of the labial mask. Indeed, the average velocity of the return of the labial mask to its initial position is a strong evidence of the effect of temperature on the muscle contraction in the labial mask of *A. imperator*. The reconciliation of these diverging results lies probably in morphological differences in the apparatus for capturing prey having different thermal sensitivity (Scales, O'Donnell, & Deban, 2017). For example, the tongue projection of ballistic and nonballistic salamanders has different thermal sensitivity because they have different mechanisms: elastically and muscle powered, respectively, and the muscle powered being more sensitive to temperature (Scales et al., 2017). Similar patterns were also found for some species of frogs (Sandusky & Deban, 2012). This effect might explain why temperature has a negative effect on the survival rate of prey with some dragonfly species and not with others (Eck, Byrne, Popescu, Harper, & Patrick, 2014). To our knowledge, there is no study identical to ours on the effect of temperature on different species and groups of Odonata larvae (e.g., Zigoptera and Anisoptera) which have morphologically different labial masks (Büsse, Hörnschemeyer, & Gorb, 2017; Tanaka & Hisada, 1980). Analyzing temperature effects on the attack kinematics among the species of Odonata might uncover different warming sensitivities of these key predators.

To conclude, while we found that temperature increases the velocity of the attack, this effect does not translate necessarily in an increase in success rate for the dragonfly. The reverse is true too: Temperature increase does not increase the failure of the attack via inaccuracy. Thus, the speed–accuracy trade‐off does not seem to apply to this type of predator–prey system with a fast attack strategy of the predator. This system adds up to other cases for which the speed–accuracy trade‐off was not verified either, like in sticklebacks (Mamuneas, Spence, Manica, & King, 2015). We cannot exclude, however, that a speed–accuracy compromise applies during the prey‐detection phase of the predator, before the launch of the fast attack. The large influence of such biological traits or life histories might explain the complexity of the relationships between temperature, predators, and their prey (Rall et al., 2010). Comparative studies on how predators compute and target they prey, such as those carried out with dragonfly adults (Lin & Leonardo, 2017), tiger beetles (Gilbert, 1997; Haselsteiner, Gilbert, & Wang, 2014), or preying mantis (Rossel, 1980), as function of temperature should help resolving these issues. Other organisms have developed convergent prey capture strategies, such as the rover beetles of the genus *Stenus* which use an extended labium with sticky substances to capture jumping collembolan (Betz, 2002). A comparison of the different capturing tactics and strategies, and their efficiency at different temperatures, ranging from these insects to vertebrates such as archer fishes and chameleons, would give new insights into comparative biomechanics under the influence of temperature.

## CONFLICT OF INTEREST

None declared.

## AUTHOR CONTRIBUTIONS

E. Quenta contributed to the sample collection during fieldworks, experimental manipulation, data recording and statistical analysis, writing of the manuscript, hypothesis and scientific ideas on temperature effect on prey–predator interaction. J. Casas contributed to writing and revision of the manuscript and innovative scientific ideas on the ethology, neuroethology, and biomechanics of the organisms during the predator–prey interaction. O. Dangles contributed to significant inputs during project conception, design, and manuscript writing. S. Pincebourde contributed to the sample collection during fieldworks, writing and revision of the manuscript and innovative scientific ideas on temperature effect on prey–predator interaction.
