# Supplementary material for: Temperature effects on ballistic prey capture by a dragonfly larva
Source: Ecol Evol. 2018 Apr 2;8(8):4303–11. doi: 10.1002/ece3.3975 (PMC5916278; doi:10.1002/ece3.3975)
Supplement: Supplementary file 1 [file ECE3-8-4303-s001.docx]

**Appendices**

**Appendix 1.** Experimental design

**Table S1.** Number of films selected from the first group of films (2000 images/second) at the three temperature treatments (15ºC, 20 ºC and 30 ºC). The actual water temperature during each film is reported.

| **Treatment** | **Temperature (ºC)** | **Individuals (ID)** | **Number of films selected** |
| --- | --- | --- | --- |
|  | 15.1/15.4 | Larvae#1 | 2 |
|  | 14.9/15.0/14.6 | Larvae#2 | 3 |
| **15 ºC** | 15.1/14.6/15.6/15.0 | Larvae#3 | 4 |
|  | 15.1/15.1/15.1/15.3 | Larvae#4 | 4 |
|  | 21.3/20.1/21.1/20.5 | Larvae#5 | 4 |
| **20ºC** | 20.3/20.0/20.1/19.1/19.0 | Larvae#6 | 5 |
|  | 20.8/20/19.8/21.5/20.1 | Larvae#7 | 5 |
|  | 18.1/20.4/20.8 | Larvae#8 | 3 |
|  | 30.0/30.1/30.4 | Larvae#9 | 3 |
|  | 29.9/30.2/30.0/29.8 | Larvae#10 | 4 |
| **30ºC** | 30.2/29.9/30.0/29.9/30.0 | Larvae#11 | 5 |
|  |  |  | **Total = 42 films** |

| **Treatment** | **Temperature (ºC)** | **Individuals (ID)** | **Number of films** | **Number of attacks per film** |
| --- | --- | --- | --- | --- |
| **15ºC** | 14.4 / 15.4 / 15.6 | Larvae#2 | 3 | 3 / 1 / 1 |
|  | 15.1 / 14.9 / 14.8 / 15 / 15 / 15 / 15 | Larvae#3 | 7 | 2 / 1 / 2 / 1 / 1 / 1 / 1 |
|  | 15.4 / 15.2 / 15.4 / 14.5 | Larvae#4 | 4 | 1 / 1 / 1 / 1 |
| **20ºC** | 20.8 / 19.6 / 19.9 / 19.9 / 20 / 20 / 20.1 | Larvae#5 | 7 | 1 / 1 / 2 / 1 / 1 / 4 / 1 |
|  | 18.7 / 19.1 / 20.3 / 20.3 | Larvae#6 | 4 | 1 / 1 / 2 / 1 |
|  | 20.3 / 20.4 / 20.2 / 20 / 20.6 / 20.3 | Larvae#7 | 6 | 1 / 1 / 3 / 3 / 1 / 2 |
|  | 18.1 / 18.6 / 20.2 / 20.3 / 20.5 / 20.6 | Larvae#8 | 6 | 1 / 1 / 1 / 1 / 1 / 1 |
| **30ºC** | 30.6 / 30.2 / 30.6 | Larvae#9 | 3 | 1 / 1 / 2 |
|  | 29.6 / 29.5 / 30.2 / 30.2 / 30.1 / 29.9 / 29.8 | Larvae#10 | 8 | 1 / 1 / 1 / 1 / 1 / 3 / 1 / 1 |
|  | 29.9 / 30.1 / 30 / 29.9 / 30 / 30 | Larvae#11 | 6 | 1 / 1 / 3 / 6 / 1 / 2 |
|  |  |  | **Total = 54** |  |

**Table S2.** Number of films selected from the second group of films (100 images/second) at the three temperature treatments (15 ºC, 20 ºC and 30 ºC). For each individual, the actual temperature during each subsequent sequence is reported, as well as the number of attacks during each of these sequences.

**Appendix 2.**

**Fig. S2.** Distance of the labial mask of the predator over time with standardized values (distance values divided by the maximum). The curves show that the movement of individuals had the same pattern during the attack. The different colors represent the temperature treatments: black=15ºC, orange=20ºC and red=30ºC.


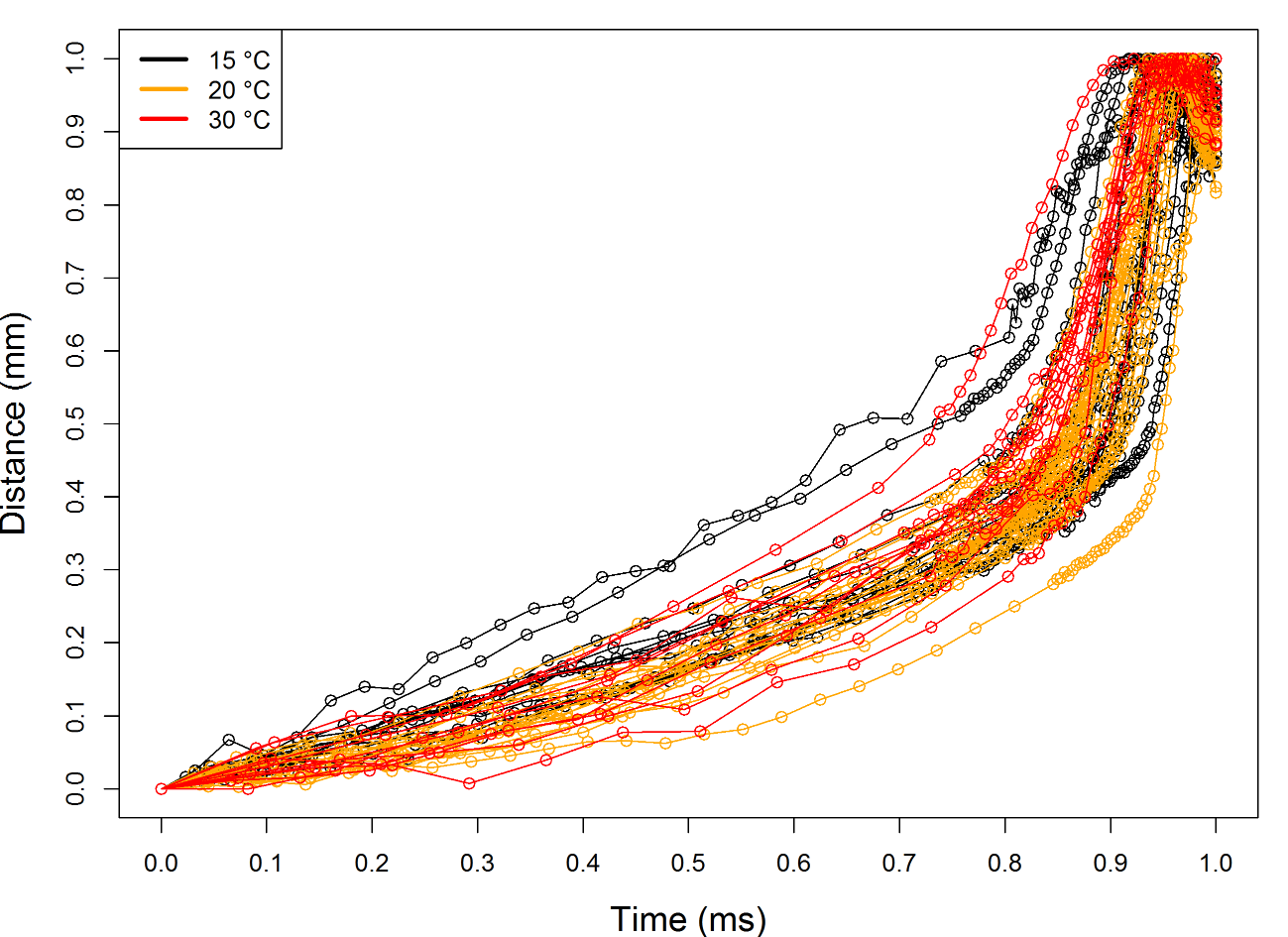


**Appendix 3.** Multiple regression analysis to test the effect of temperature, size of the labial mask (postmentum), abdomen size and body size on the velocity of the labial mask. Analyses of deviance were run to AIC values to rank the importance of each variable.

| **Model** | **Effect** | **Estimate** | **P-value** | **F value** | **AIC** |
| --- | --- | --- | --- | --- | --- |
| 1. Average velocity ~ Temperature+Postmentum size+ Temperature* Postmentum size | Intercept  Temperature  Postmentum size  Temperature* Postmentum size | -0.00178  0.008  0.006  0.00095 | **<0.001**  0.545  0.627 | 41.46  0.404  0.25 | -26.351  -47.017  -47.239 |
| 1. Average velocity ~ Temperature+Abdomen size+ Temperature* Abdomen size | Intercept  Temperature  Abdomen size Temperature* Abdomen size | -0.008 0.0075  0.002  0.0006 | **<0.01**  0.37  0.22 | 29.17  0.88  1.76 | -81.39  -81.3  -80.23 |
| 1. Average velocity ~ Temperature+Body size+ Temperature* Body size | Intercept  Temperature  Body size  Temperature* Body size | -0.010 0.0063  0.0034  0.0005 | **< 0.01**  0.085  0.085 | 23.00  3.99  4.095 | -69.93  -80.98  -80.87 |

**Appendix 4.** Parameters of the linear mixed-effects models. The significant values for each individual model was estimated by comparing to their corresponding null model: velocity~1+(1|larva).

| **Model** | **Fixed effects** | **Estimate** | **SE** | **P-value** | **X^2^** |
| --- | --- | --- | --- | --- | --- |
| 1. Average Velocity Forward ~ Temperature + (1 \| larva) | Intercept  Temperature | -0.04  0.0076 | 0.023  0.001 | **<0.001** | 17.98 |
| 1. Average Velocity return ~ Temperature + (1 \| larva) | Intercept  Temperature | -0.093 0.0097 | 0.022  0.001 | **<0.001** | 26.89 |
| 1. Average Initial Velocity ~ Temperature + (1 \| larva) | Intercept  Temperature | -0.035  0.0051 | 0.016  0.0007 | **<0.001** | 18.78 |
| 1. Average Final Velocity ~ Temperature + (1 \| larva) | Intercept  Temperature | 0.40  0.017 | 0.18  0.084 | **<0.05** | 4.26 |

**Appendix 5.**

**Fig. S5. N**umber of attacks at each temperature treatment. No significant effect was found between the temperature and the number of attacks by the predators in a regression analysis (χ^2^= 1.10, P=0.29).


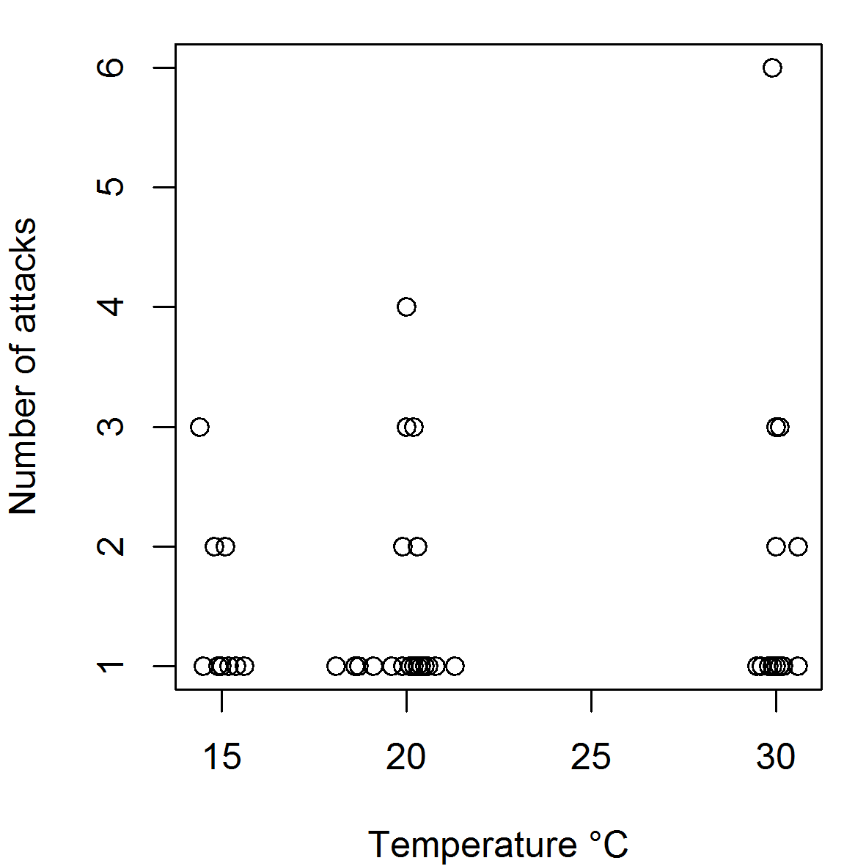


**Appendix 6. G**eneralized linear mixed model (GLMM) analysis of the probability of capture success. The parameters were estimated by their maximum likelihood using Laplace approximations, chi-squared tests, and the Akaike information criterion (AIC), following the procedure of (Bolker et al., 2009; Bates et al., 2015). Parameters were transformed to probability values with the *plogis* function using the *lme4* R package.

| **Model** | **Fixed effects** | **Estimate** | **Probability** | **P-value** | **X^2^** | **AIC** |
| --- | --- | --- | --- | --- | --- | --- |
| 1. Capture to prey ~ Distance Prey Predator + (1 \| larva) | Intercept  Distance | 5.43  -0.74 | **0.995 (99.5%)**  **0.388 (38.8%)** | **<0.05** | 5.31 | 52.55 |
| 1. Model1+Temperature   + (1 \| larva) | Intercept  Temperature | -0.59  0.06 | 0.95  0.52 | 0.201 | 1.62 | 52.92 |
| 1. Model2+   Distance*Temperature  + (1 \| larva) | Intercept  Distance*Temperature | 3.16  0.11 | 0.00005  0.48 | 0.10 | 2.65 | 52.27 |
| 1. Model1+Initial Velocity + (1 \| larva) | Intercept  Initial velocity | 3.98  12.55 | 0.98  0.99 | 0.34 | 0.88 | 53.66 |
| 1. Model4+   Distance* initial velocity  + (1 \| larva) | Intercept  Distance*Initial velocity | -0.09  -6.64 | 0.476  0.001 | 0.26 | 1.26 | 54.40 |
| 1. Model1+Final Velocity   + (1 \| larva) | Intercept  Final Velocity | 4.8  1.15 | 0.99  0.760 | 0.58 | 0.29 | 54.25 |
| 1. Model6+ Distance* Final velocity   + (1 \| larva) | Intercept  Distance* Final velocity | 5.18  0.043 | 0.994  0.51 | 0.94 | 0.0056 | 56.25 |
| 1. Model1+ Medium velocity   + (1 \| larva) | Intercept  Medium velocity | 4.18  8.14 | 0.98  0.99 | 0.36 | 0.80 | 53.71 |
| 1. Model8+ Distance* Medium velocity   + (1 \| larva) | Intercept  Distance* Medium velocity | -0.38  -4.64 | 0.40  0.0095 | 0.19 | 1.71 | 54.032 |
